# Cilia and sensory signaling: The journey from “animalcules” to human disease

**DOI:** 10.1371/journal.pbio.2002240

**Published:** 2017-04-14

**Authors:** Piali Sengupta

**Affiliations:** Department of Biology and National Center for Behavioral Genomics, Brandeis University, Waltham, Massachusetts, United States of America

## Abstract

Nearly all cell types in mammals contain cilia, small rod-like or more elaborate structures that extend from the cell surface. Cilia house signaling proteins that allow the cell to sample their environment and respond appropriately. Mutations in ciliary genes alter the functions of a broad range of cell and tissue types, including sensory and central neurons, and underlie a collection of heterogeneous human disorders called ciliopathies. Here, I highlight the critical contributions of nearly three centuries of research in diverse organisms to our current knowledge of cilia function in sensory signaling and human disease.

In the late 17th century, the tradesman and amateur microscopist Antonie van Leeuwenhoek described motile “thin little feet, or little legs” on what he referred to as “animalcules.” We now know that he was looking at cilia on what were likely ciliated protozoa (see [1] for an excellent historical account; Fig 1). Little did van Leeuwenhoek know that cilia in unicellular organisms would hold the key to our understanding of the mechanisms underlying a plethora of debilitating human disorders—a connection scientists would make nearly three centuries later. In one of many examples of our inability to foresee the ultimate importance of seemingly esoteric lines of inquiry, it took the work of scientists studying the motile flagella of an alga to link the work of Leeuwenhoek with that of modern developmental and cell biologists, neuroscientists, and clinicians to uncover the critical roles of cilia in regulating metazoan physiology.

**Fig 1:**
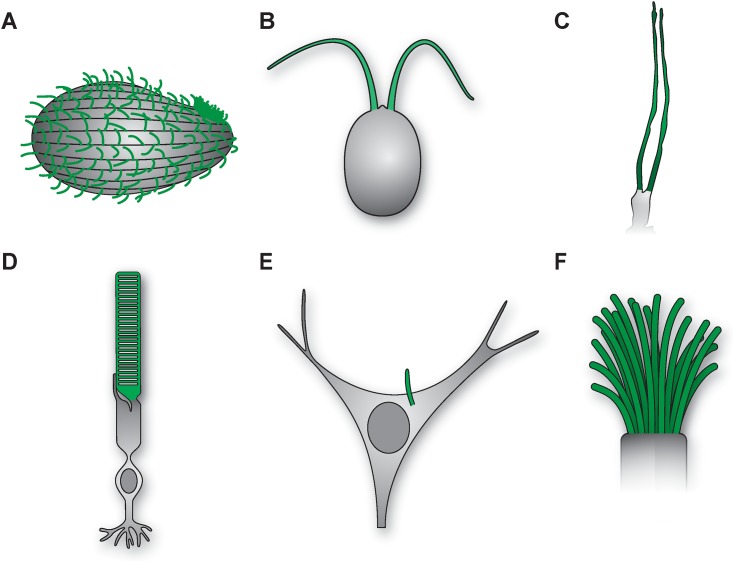
Cilia are sensory organelles that are found in multiple species and are present on nearly all cell types in mammals. Schematics of cilia/flagella (green) in the ciliated protozoa *Tetrahymena* (A), the alga *Chlamydomonas* (B), the *Caenorhabditis elegans* sensory neuron ADL (C), a mammalian rod photoreceptor (D), a mammalian hypothalamic neuron (E), and a mammalian airway epithelial cell (F). Drawings are not to scale. Drawing by Julian Eskin.

In 1993, Keith Kozminski, Joel Rosenbaum, and colleagues studying flagellar motility in the green alga *Chlamydomonas* (Fig 1) described a protein transport process that they termed intraflagellar transport (IFT) [2]. Rosenbaum and colleagues went on to show that IFT is mediated by a large protein complex and identified many of the proteins that make up this complex [3]. The majority of these proteins are essential for transporting structural and signaling molecules within the flagella; mutations in IFT protein genes disrupted flagella and rendered these algae immobile. Remarkably, it was found that IFT genes are extremely well conserved across eukaryotes, including in humans, suggesting that these genes were present in the last common eukaryotic ancestor.

One of the first indications that IFT genes can be linked with disease in mammals came from Greg Pazour and colleagues, who showed that mice carrying mutations in one of the homologs of the *Chlamydomonas* IFT genes exhibited polycystic kidney disease [4]. How can the homolog of a protein required to build *Chlamydomonas* flagella regulate mouse and human kidney function? Interestingly, the answer to this question had also been intimated over a century ago. In 1876, in a series of beautiful drawings, Paul Langerhans had described cilia-like structures on multiple cell types of the primitive chordate *Amphioxus* [1]. In 1898, Karl Wilhelm Zimmermann noted similar structures on mammalian cells, including kidney cells, and made the prescient observation that these structures likely transmit sensory information to the cell [1]. Indeed, we now know that disruption of these structures on kidney cells leads to renal disease. Although these studies were largely forgotten for a period of time, advances in transmission electron microscopy led to the realization that cilia, with some structural diversifications, are present on nearly every cell type in vertebrates.

Cilia can be categorized into two general classes: motile and immotile. Motile cilia are found on cells such as those lining the airways in humans (Fig 1), but the majority of cells contain immotile cilia, which are now referred to as primary cilia. Both motile and primary cilia are built via similar IFT-based mechanisms. (Flagella present on *Chlamydomonas* and human sperm are essentially motile cilia, and the terms cilia and flagella are now used interchangeably). As postulated by Zimmermann, cilia have been established to be sensory organelles [5] and are responsible for sensing and transducing environmental signals to maintain cellular homeostasis. The finding that cilia dysfunction affects multiple tissue types in humans in turn led to the characterization of the molecular mechanisms underlying a genetically and phenotypically heterogeneous set of diseases in humans, collectively termed ciliopathies [6]. In fact, nearly all genes implicated in ciliopathies have been shown to regulate the structure and function of cilia across species.

As sensory neuroscientists, members of my lab and I have focused for the past two decades on identifying and describing the molecular and neuronal mechanisms by which animals sense and respond reliably, yet flexibly, to environmental signals. Primary cilia play a critical role in brain development [7], although the functions of these organelles in the mature brain remain to be fully elucidated. Nonetheless, anatomists and neuroscientists have long been aware that cilia are essential for the functions of sensory neurons, such as olfactory neurons and photoreceptors present in the olfactory epithelia of the nose and in the eye, respectively (Fig 1). These neurons contain specialized cilia at their dendritic endings that house signal transduction molecules required to sense and respond to environmental cues such as chemicals or photons. Indeed, ciliary localization of these molecules in part underlies the remarkable sensitivity and broad dynamic response range of these neurons. The crucial role of cilia in regulating sensory neuron function is highlighted by the fact that many ciliopathies are characterized by sensory disorders, including retinal degeneration, loss of the ability to smell (anosmia), and deafness [6].

Our experimental system is the nematode *Caenorhabditis elegans*, an animal that not only exhibits extremely robust and complex sensory behaviors that can be quantified at high resolution but also offers a powerful array of experimental tools for manipulating gene and neuronal functions. The basic principles of sensory signal transduction apply across modalities and species, even if individual molecules (such as olfactory receptors) may have evolved in a species- and niche-specific manner [8]. As in mammals, sensory signaling molecules in *C*. *elegans* are concentrated in specialized cilia at the dendritic endings of sensory neurons (Fig 1). However, unlike in mammals, only sensory neurons are ciliated in this organism, and worms can survive in the laboratory without cilia. This is a major experimental advantage because complete loss of cilia in mammals results in embryonic lethality, partly due to the loss of cilia-dependent signaling by Sonic hedgehog (Shh), a major developmental morphogen [9]. As a consequence, we have been able to address questions related to cilia biology in this organism that are more challenging to address in mammals.

Using this model organism, we are identifying genetic pathways that regulate the development and maintenance of elaborate sensory neuron-specific ciliary architectures, describing mechanisms that localize sensory signaling proteins to specific subdomains of these cilia, exploring how signaling protein organization in cilia contributes to neuronal properties, and dissecting the intricate feedforward and feedback signaling between sensory signaling and cilia structure [10–13]. Many of the molecules and signaling principles identified in *C*. *elegans* are conserved and have been implicated in regulating sensory neuron and cilia function in vertebrates. By combining behavioral neurogenetics with cilia cell biology, we expect to be able to provide a more nuanced and complete description of sensory signal transduction mechanisms, which will help us elucidate how disruption of sensory signaling and cilia function affects animal development and behavior. Our hope is that the work of my lab and that of my colleagues studying cilia biology in multiple cellular contexts in organisms as diverse as *Chlamydomonas*, *Tetrahymena*, *Paramecium*, *Drosophila*, *C*. *elegans*, zebrafish, frogs, mice, and of course human cells [14, 15] will not only allow us to satisfy our general curiosity as to “how things work” in these different organisms but also enable us to eventually derive strategies for targeting ciliopathies.

Making the connection between Leeuwenhoek’s little legs on protists with primary cilia on metazoan cells took nearly three centuries of seemingly disparate research avenues in organisms residing on far-flung branches of the eukaryotic tree. Science is rife with many similar examples of once obscure observations providing the foundation for major scientific breakthroughs. While basic research may not be our only hope (with apologies to Princess Leia), with some patience, it is perhaps our best hope for understanding the basis for many human diseases.
